# Molecular and functional characterization of the *BMPR2* gene in Pulmonary Arterial Hypertension

**DOI:** 10.1038/s41598-017-02074-8

**Published:** 2017-05-15

**Authors:** Guillermo Pousada, Vincenzo Lupo, Sheila Cástro-Sánchez, María Álvarez-Satta, Ana Sánchez-Monteagudo, Adolfo Baloira, Carmen Espinós, Diana Valverde

**Affiliations:** 1 Dep. Biochemistry, Genetics and Immunology. Faculty of Biology, University of Vigo, As Lagoas Marcosende S/N, 36310 Vigo, Spain; 2 Grupo de Investigación Enfermedades Raras y Medicina Pediátrica, Instituto de Investigación Sanitaria Galicia Sur (IIS Galicia Sur), SERGAS-UVIGO, Vigo, Spain; 3 Unit of Genetics and Genomics of Neuromuscular and Neurodegenerative Disorders, Centro de Investigación Príncipe Felipe (CIPF), 46012 Valencia, Spain; 4 Neumology Service, Complexo Hospitalario Universitario de Pontevedra, 36071 Pontevedra, Spain

## Abstract

Pulmonary arterial hypertension is a progressive disease that causes the obstruction of precapillary pulmonary arteries and a sustained increase in pulmonary vascular resistance. The aim was to analyze functionally the variants found in the *BMPR2* gene and to establish a genotype-phenotype correlation. mRNA expression studies were performed using *pSPL3* vector, studies of subcellular localization were performed using *pEGFP-N1* vector and luciferase assays were performed using *pGL3-Basic* vector. We have identified 30 variants in the *BMPR2* gene in 27 of 55 patients. In 16 patients we detected pathogenic mutations. Minigene assays revealed that 6 variants (synonymous, missense) result in splicing defect. By immunofluorescence assay, we observed that 4 mutations affect the protein localization. Finally, 4 mutations located in the 5′UTR region showed a decreased transcriptional activity in luciferase assays. Genotype-phenotype correlation, revealed that patients with pathogenic mutations have a more severe phenotype (sPaP p = 0.042, 6MWT p = 0.041), a lower age at diagnosis (p = 0.040) and seemed to have worse response to phosphodiesterase-5-inhibitors (p = 0.010). Our study confirms that *in vitro* expression analysis is a suitable approach in order to investigate the phenotypic consequences of the nucleotide variants, especially in cases where the involved genes have a pattern of expression in tissues of difficult access.

## Introduction

Pulmonary arterial hypertension (PAH; OMIM #178600, ORPHA 422) is a rare, progressive disease that typically causes the obstruction of precapillary pulmonary arteries. It is characterized by a sustained increase in mean pulmonary artery pressure (mPaP) ≥ 25 mmHg at rest with normal pulmonary arterial wedge pressure (PAWP) ≤ 15 mmHg^2^. Syncope, dyspnea and chest pain are the main symptoms of PAH, which eventually lead to premature death due to right sided heart failure^1^. In addition, an increase in pulmonary vascular resistance (PVR) is observed in these patients, mainly due to both thrombus formation and structural and functional changes in the vascular wall^2^. Mean age at presentation, ranges from 36 to 50 years in adults, although individuals at any age can be affected^2, 3^. This pathology is more frequent in women, with a ratio of 1.7:1 women to men^3, 4^. PAH is classified as idiopathic (IPAH), hereditary (HPAH) or associated with other conditions (APAH) such as connective tissue diseases, congenital heart diseases, portal hypertension and drug or toxin exposure^5, 6^. When a genetic defect has been identified in IPAH patients, which cosegregates with disease, they have been classified as HPAH^7^.

Regarding to the genetic basis of PAH, the main gene involved is bone morphogenetic protein receptor type 2 (*BMPR2*), located on chromosome 2q33. Mutations in this gene have been identified in more than 80% of patients with HPAH, although only 20% of carriers eventually develop the disease^4, 8–11^. On the other hand, the frequency of *BMPR2* mutations in IPAH patients is much lower, ranging from 6–40%^12–15^. *BMPR2* encodes for a transmembrane serine/threonine kinase receptor belonging to the transforming growth factor beta (TGF-β) superfamily, and is specifically recognized by bone morphogenetic proteins (BMPs), which are involved in several signalling pathways that regulate cellular differentiation, proliferation and apoptosis^16, 17^. Either loss of function or reduction in *BMPR2* expression may be sufficient to develop PAH^16^.

Mutational screening of *BMPR2* in PAH patients has been extensively reported^9–11, 13^. However, little is known about the real pathogenicity of missense, synonymous or intronic changes, among others, in the PAH development. Thus, it is well established that synonymous and also non-synonymous variants can affect the conformation and stability of mRNA, the splicing process, the accuracy of translation and the protein structure^17^. Moreover, splicing mutations represent more than 9% of the published changes, although experimental confirmation should increase the percentage of splicing mutations^18^. In this sense, the minigene assays have been reported as a good approach to evaluate potential splicing alterations produced by these variants of uncertain pathogenicity^19^, especially when the gene expression profile is restricted and/or patient′s tissue samples are difficult to obtain, as in PAH. On the other hand, it is well known that mutations can lead to disease because the protein gets mislocalized and therefore, cannot carry out its activity properly^20^. Finally, the role of the *BMPR2* variants in promoter region has been scarcely characterized, but known to affect gene expression^21^.

Taking into account the importance of performing functional studies in order to determine if a variant is pathological, our main objectives were: (1) to investigate if the detected *BMPR2* mutations could be associated with alterations in mRNA processing, subcellular localization and/or transcriptional activity and therefore, they could cause an abnormal protein activity; and (2) to establish an accurate genotype-phenotype correlation, comparing the set of clinical and hemodynamic features of patients harbouring pathogenic mutations with those without them.

## Results

### Description of the cohort

**Figure 1:**
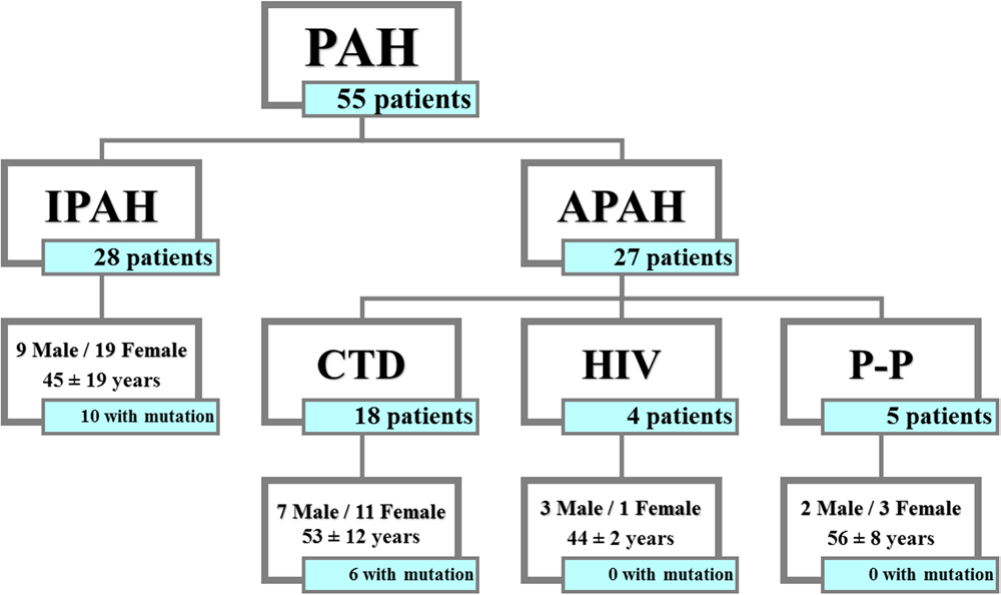
Diagram of the population under study. This figure shows the total number of patients included in this study (55 patients) classified according to PAH type (28 patients with IPAH and 27 patients with APAH), gender, mean age at diagnosis and pathogenic mutations in *BMPR2* gene. PAH: Pulmonary Arterial Hypertension; IPAH: Idiopathic Pulmonary Arterial Hypertension; Associated Pulmonary Arterial Hypertension; CTD: Connective Tissue Disease; HIV: Human Immunodeficiency Virus; P-P: Porto-Pulmonary Hypertension.

This cohort has been described previously by our group and therefore, mutations in the *BMPR2*, *ACVRL1* (Activin A type II receptor like kinase 1), *ENG* (Endoglin) and/or *KCNA5* (Potassium voltage-gated channel, shakerrelated subfamily, member 5) genes have been previously reported by Pousada *et al*.^10, 22, 23^. In summary, 55 unrelated PAH patients of Spanish origin (28 IPAH, 18 APAH associated to connective tissue disease, 4 related to HIV and 5 porto-pulmonary hypertension) (Fig. 1) and 50 healthy controls without familial history of PAH were included. At the time of diagnosis 7 patients were in functional class (FC) I, 19 patients in FC II, 25 patients in FC III and 4 in FC IV. Thirty-three out of the 39 patients were considered responders to treatment, compared to only 2 out of 16 patients with pathogenic mutations. There was a decrease in proBNP of 482 ± 292 pcg/mL in responders after 6 months of treatment with significant differences regarding non-responders (115 ± 384 pcg/mL, p < 0.001). Clinical features of patients are shown in Table 1.

**Table 1:** Clinical features and hemodynamic parameters of patients included in this study.

Clinical features and hemodynamic parameters	Total patients	Patients with proven pathogenic mutations	
		Clinical data	p-value*
*Number*	55	16	—
*Gender*	20 M/35 F	4 M/12 F	0.360
*Age at diagnosis* (*years*)	49 ± 16	38 ± 16	0.040
*mPaP* (*mmHg*)	49 ± 14	47 ± 6	0.266
*sPaP* (*mmHg*)	70 ± 19	60 ± 9	0.042
*PVR* (*mmHg.l* ^−*1*^ *.m* ^−1^)	7.2 ± 3.3	7.9 ± 0.5	0.553
*CI* (*l.m* ^*−1*^ *.m* ^−*2*^)	2.5 ± 0.7	2.3 ± 0.3	0.588
*6MWT* (*m*)	415 ± 146	570 ± 86	0.041
*proBNP* (*pcg*/*mL*)	1276 ± 434	1297 ± 465	0.526
*PAH types*	28 IPAH/27 APAH	10 IPAH/6 APAH	0.137
*No improvement upon treatment*	22	14	0.010

Values are expressed as mean ± standard deviation; F: female, M: male; mPaP: mean pulmonary artery pressure; sPaP: systolic pulmonary artery pressure; PVR: pulmonary vascular resistence; CI: cardiac index; 6MWT: 6 minute walking test; IPAH: idiopathic pulmonary arterial hypertension; APAH: associated pulmonary arterial hypertension.

*Clinical features and hemodynamic parameters among patients with pathogenic changes in the *BMPR2* gene and without.

### Mutational analysis of the *BMPR2* gene

**Figure 2:**
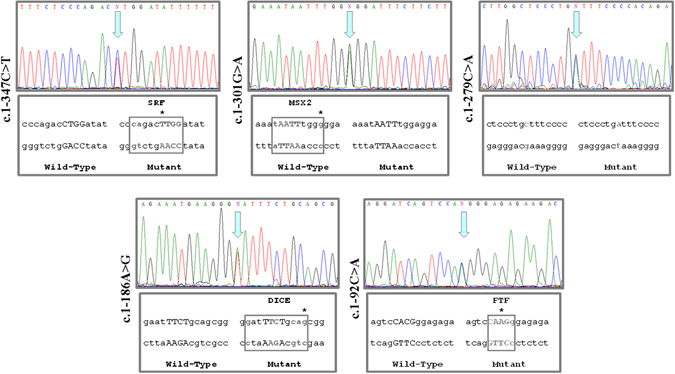
Representative sequence electropherograms for the mutations in 5′UTR of *BMPR2* gene (c.1-347C > T, c.1-301G > A, c.1-279C > A, c.1-186A > T and c.1-92C > A) in PAH patients and *In silico* analysis of the effect on the binding site to transcriptions factors of variants found in 5′UTR region of *BMPR2* gene. MatInspector software found that these variants could create (SRF, DICE and FTF) or remove (MSX2) new binding sites for different factors. c.1-279C > A seems to not the binding sites to transcriptions factors.

The molecular analysis of the 5′UTR region (539 bp) of the *BMPR2* gene revealed 5 nucleotide changes in 6 patients (11%), all of them in heterozygous state (Fig. 2). All but one were females. Four patients had IPAH and one patient presented with PAH associated with systemic sclerosis. These 5 mutations were absent in a panel of 100 control chromosomes. However, c.1-301G > A (rs116154690) mutation was found in the Ensembl Database with a 0.01 minor allele frequency (MAF). The *in silico* analysis predicted a negative impact on the splicing process for these variants, as expected (Table 2).

**Table 2:** *In silico* analysis of variants identify in the *BMPR2* gene.

Nucleotide change	PolyPhen-2^74^	Pmut^75^	Sift^76^	Mutation Taster^77^	NNSplice^78^	NetGene2^78^	Splice View^78^	HSF^79^
c.1-347C > T	—	—	—	—	The WT consensus sequence is not recognized	Score for the main donor site increases	The WT consensus sequence is not recognized	Neutral
c.1-301G > A	—	—	—	—	The WT consensus sequence is not recognized	Score for the aceptor site increases	The WT consensus sequence is not recognized	Neutral
c.1-279C > A	—	—	—	—	The WT consensus sequence is not recognized	Score for the main donor site increases	The WT consensus sequence is not recognized	Neutral
c.1-186A > T	—	—	—	—	The WT consensus sequence is not recognized	Score for the main donor site increases	The WT consensus sequence is not recognized	Neutral
c.1-92C > A	—	—	—	—	The WT consensus sequence is not recognized	Score for the main donor site increases	The WT consensus sequence is not recognized	Neutral
c.156_157delTC (p.S52Sfs*1)	—	—	—	—	Neutral	Score for the main donor site and the main acceptor site decreases	The WT consensus sequence is not recognized	A new acceptor site is created
c.190A > C (p.S64R)	Benign	Neutral	Tolerated	Disease causing	Neutral	The WT consensus sequence is not recognized	A new donor site is created	Score for donor and acceptor site decreases
c.229A > T (p.I77L)	Benign	Neutral	Damaging	Disease causing	The WT consensus sequence is not recognized	Score for the main donor site increases	Neutral	A new acceptor site is created
c.251G > T (p.C84F)	Probably damaging	Neutral	Damaging	Disease causing	Score for the acceptor site increases	Score for the main acceptor site decreases	Neutral	The main donor site is not recognized
c.259C > T (p.H87Y)	Benign	Neutral	Damaging	Disease causing	Score for the acceptor site decreases	Score for the main acceptor site decreases	Neutral	The main donor site is not recognized and the acceptor
c.275A > T (p.Q92L)	Benign	Pathologic	Damaging	Disease causing	Neutral	Score for the main acceptor site decreases	Neutral	Score for donor and acceptor site increases
c.327G > A (p.Q109Q)	—	—	—	—	Neutral	Score for the main donor site decreases	Neutral	The main donor site is not recognized
c.327G > C (p.Q109H)	Probably damaging	Neutral	Tolerated	Disease causing	Neutral	Score for the main donor site decreases	Neutral	The WT consensus sequence is not recognized
c.412C > G (p.P138A)				Disease causing	Neutral	Neutral	Neutral	Neutral
c.484G > C (p.A162P)	Probably damaging	Neutral	Damaging	Disease causing	Score for the acceptor site decreases	Neutral	Neutral	The main donor site is not recognized
c.600A > C (p.L200L)	—	—	—	—	Neutral	Neutral	Neutral	Neutral
c.633A > G (p.R211R)	—	—	—	—	Neutral	Score for the main donor site increases	Neutral	The main donor site is not recognized and the acceptor decrease
c.637C > A (p.R213R)	—	—	—	—	Neutral	Score for the main acceptor site decreases	Neutral	Score for donor site increases and a new acceptor site is created
c.654T > A (p.Y218*)	—	—	—	—	Neutral	Score for the main donor site increases and the main acceptor site decreases	Neutral	Score for the main acceptor site decrease
c.742A > G (p.R248G)	Benign	Pathologic	Damaging	Disease causing	Neutral	Score for the main donor site decreases	Neutral	Score for the main donor site and the main acceptor site increases
c.790G > A (p.D264N)	Possibly damaging	Neutral	Damaging	Disease causing	Neutral	Score for the main donor site decreases	Neutral	The main donor site is not recognized
c.835G > T (p.V278V)	—	—	—	—	Neutral	Neutral	Neutral	Score for donor site decreases and the acceptor site increase
c.893G > A (p.W298*)	—	—	—	—	Neutral	Score for the main donor and acceptor site decreases	The WT consensus sequence is not recognized	The main donor site increase and a new acceptor site in created
c.981T > > > C (p.P327P)	—	—	—	—	The WT consensus sequence is not recognized	Score for the main donor site decreases	Neutral	A new donor site is created
c.1021G > A (p.V341M)	Possibly damaging	Neutral	Damaging	Disease causing	Neutral	Neutral	The WT consensus sequence is not recognized	The main donor site is not recognized
c.1400A > G (p.K467R)	Possibly damaging	Neutral	Damaging	Disease causing	Neutral	Score for the main donor site increases	Neutral	The main donor site is not recognized
c.1467G > A (p.E489E)	—	—	—	—	Neutral	Score for the main donor site increases	Neutral	A new acceptor site is created
c.2324G > A (p.S775N)	Benign	Neutral	Tolerated	Polymorphism	Neutral	Neutral	Neutral	The main donor site is not recognized

These mutations, located in the 5′UTR region, could affect the binding site for several transcription factors. Thus, c.1-347C > T may create a new binding site for SRF (*Serum response factor*), c.1-186A > T for DICE (*Downstream Immunoglobulin Control Element*), and c.1-92C > A for FTF (*Alpha (1)-fetoprotein transcription factor*). The c.1-301G > A change could delete the binding site for MSX2 (*Muscle-segment homebox 2*) and c.1-279C > A did not produce changes in the analyzed binding sites (Fig. 2).

We have found 25 different variations in the coding region in 21 patients (38%), who included 12 patients with IPAH and 9 with APAH. The highest percentage of nucleotide alterations corresponds to missense changes, which represent 71% of total variations found, followed by synonymous changes (33%) and nonsense mutations (14%). These results were partially reported in Pousada *et al*.^10^.

All the variations identified in this study have not been found in the different databases considered, such as dbSNP (http://www.ncbi.nlm.nih.gov/SNP), ESP6500 (http://evs.gs.washington.edu/EVS/), ExAC (http://exac.broadinstitute.org/), and CSVS (http://csvs.babelomics.org/). Missense variations were analyzed *in silico* with different tools (*PolyPhen-2, Pmut, Sift and Mutation Taster*) to predict their pathogenicity and the impact on the disease. Likewise, all variations were analyzed *in silico* to know if these missense, synonymous and nonsense variations could affect donor/acceptor splice sites (*NNSplice*, *NetGene2*, *Splice View* and *Human Splice Finder (HSF)*). We classified the mutation as potentially pathogenic if at least two *in silico* tools detected any possible alteration in the canonical sequence recognition. These results are available in Table 2.

### Minigene assays

After performing an exhaustive bioinformatics analysis of the mutations identified, we detected 22 changes that presumably affect the splicing process (Tables 3 and 4). To go in depth into the possible role of these putative splicing variants, we generated minigene constructions. Our results showed that 6 out of 22 mutations (27%) have an effect on mRNA processing. Three nucleotide changes were synonymous (c.633A > G, c.835G > T and c.981T > C) and 3 were missense variants (c.251G > T, c.412C > G and c.1400A > G). Besides, we confirmed that 2 nonsense changes (c.654T > A and c.893G > A) and 1 frameshift mutation (c.156_157delTC) produce a premature stop codon, as expected.

**Table 3:** Changes with no effect on the mRNA processing.

Nucleotide change (amino acid change)	Exon	Number of patients	PAH type	Reference
**c.190A** > **C** (p.S64R)	2	1	IPAH	Pousada *et al*.^10^
**c.229A** > **T** (p.I77L)	2	1	IPAH	Pousada *et al*.^10^
**c.259G** > **T** (p.H87Y)	3	1	APAH	Pousada *et al*.^10^
**c.275A** > **T** (p.Q92L)	3	1	IPAH	Pousada *et al*.^10^
**c.327G** > **A** (p.Q109Q)	3	1	APAH	Pousada *et al*.^10^
**c.327G** > **C** (p.Q109H)	3	1	APAH	This study
**c.484G** > **C** (p.A162P)	4	1	APAH	Pousada *et al*.^10^
**c.600A** > **C** (p.L200L)	5	1	IPAH	rs55722784
**c.637C** > **A** (p.R213R)	6	1	IPAH	Pousada *et al*.^10^
**c.742A** > **G** (p.R248G)	6	1	IPAH	Pousada *et al*.^10^
**c.790G** > **A** (p.D264N)	6	1	IPAH	CD061372
**c.1021G** > **A** (p.V341M)	8	3	APAH	Pousada *et al*.^10^
**c.1467G** > **A** (p.E489E)	11	1	APAH	Pousada *et al*.^10^

**Table 4:** Pathogenic mutations found in the *BMPR2* gene in our clinical series.

Nucleotide change	Region	Score ^(1)^	Effect on protein	Number of patients	PAH type	Reference
c.1-347C > T	5′UTR	0	Expression is decreased	2	IPAH	This study
c.1-301G > A	5′UTR	0	Expression is decreased	1	APAH	rs116154690
c.1-279C > A	5′UTR	0	Expression is decreased	1	IPAH	This study
c.1-92C > A	5′UTR	0	Expression is decreased	1	IPAH	This study
c.156_157delTC	Exon 2	2	p.S52Sfs*1	1	IPAH	Pousada *et al*.^10^
c.251G > T	Exon 3	3	p.C84Ffs*12	2	APAH	Pousada *et al*.^10^
c.412C > G	Exon 3	0	p.P138_S140del	1	IPAH	This study
c.633A > G	Exon 6	2	p.E207_G212del	1	IPAH	Pousada *et al*.^10^
c.654T > A	Exon 6	2	p.Y218*	1	APAH	Pousada *et al*.^10^
c.835G > T	Exon 6	1	p.L207_N284del	1	IPAH	Pousada *et al*.^10^
c.893G > A	Exon 7	2	p.W298*	1	IPAH	Pousada *et al*.^10^
c.981T > C	Exon 8	2	p.H324_E379del	2	IPAH/APAH ^(1)^	Pousada *et al*.^10^
c.1400A > G	Exon 10	2	p.K467Rfs*27	1	IPAH	This study

(1)This mutation appears in a patient with IPAH and in a patient with APAH.

**Figure 3:**
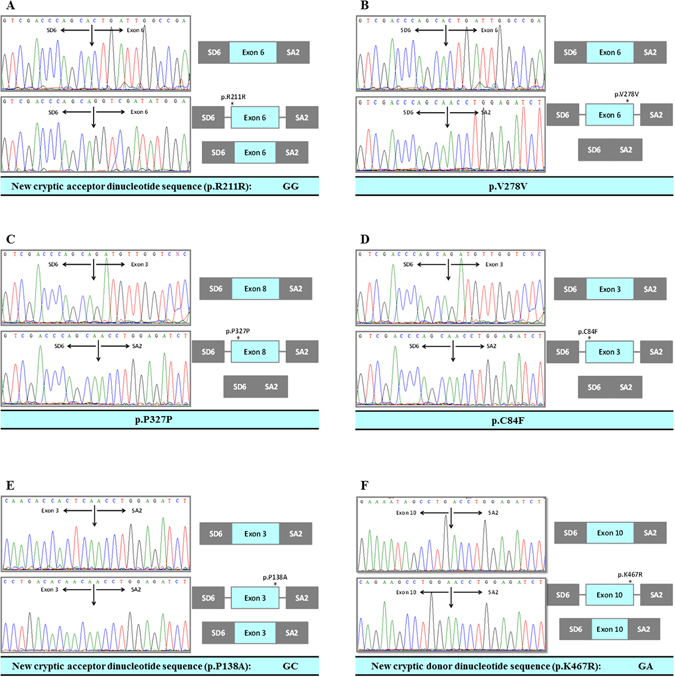
*In vitro* mRNA processing for synonymous mutations (p.R211R, p.V278V and p.P327P) and missense mutations (p.C84F, p.P138A and p.K467R) that affect the splicing process, identified in the *BMPR2* gene. The minigene assay shows the alterations caused by these mutations in mRNA processing. Three of them produces an exon skipping (p.L207_N284del, p.H324_E379del and p.C84Ffs*12) and three produces partial elimination of an exon (p.P138_S140del, p.E207_G212del and p.K467Rfs*27).

The c.633A > G mutation was first classified as a synonymous change: p.R211R. The mutant c.633A > G minigene prevented the recognition of the main acceptor site. Direct sequencing of cDNA confirmed the creation of a new 3′ alternative site, leading to a deletion of 12 bp in the 5′ region of exon 6. The mutated protein would be p.E207_G212del, which would have 1034 amino acids in length. The c.835G > T variant was first predicted as p.V278V. The construction showed that this mutation produces the skipping of exon 6, so the resulting protein (p.L207_N284del) would be 77 amino acids shorter than the wild-type BMPR2. Finally, the c.981T > C mutation, predicted first as the p.P327P protein, would cause the skipping of exon 8 and therefore, a truncated protein of 985 amino acids (p.H324_E379del) (Fig. 3A,B,C).

Mutations predicted as missense changes would modify the splicing process. Thus, c.251G > T, resulted in skipping of exon 3 and therefore, the encoded protein would have only 96 amino acids (p.C84Ffs*12). The c.412C > G mutation would produce an alternative 5′splicing site resulting in a two-amino-acid shorter protein (p.P138_S140del), since the mutation would eliminate the main donor site and would generate a new donor splice site. The analysis of c.1400A > G showed that this mutation creates a new splice donor site and therefore, the new protein would have only 494 amino acids (p.K467Rfs*27) (Fig. 3D,E,F).

**Figure 4:**
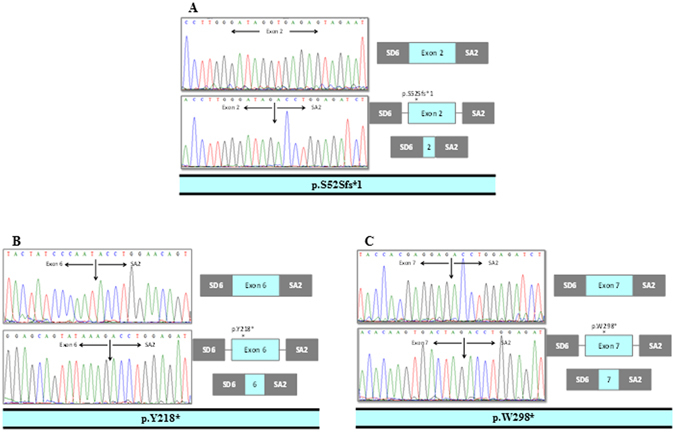
*In vitro* mRNA processing for frameshift mutation (p.S52Sfs*1) and nonsense mutations (p.Y218* and p.W298*) that produce a premature stop codon in the *BMPR2* gene, as expected.

Regarding the frameshift and nonsense mutations, they would generate shorter proteins due to premature stop codons: c.156_157delTC (p.S52Sfs*1), c.654T > A (p.Y218*) and c.893G > A (p.W298*) generating proteins of 54, 218 and 298 amino acids in length, respectively (Fig. 4A,B,C). Finally, the minigene assays showed negative results for the remaining 13 synonymous changes and therefore, their potential pathogenicity remains elusive (Table 3).

### Subcellular localization studies

**Figure 5:**
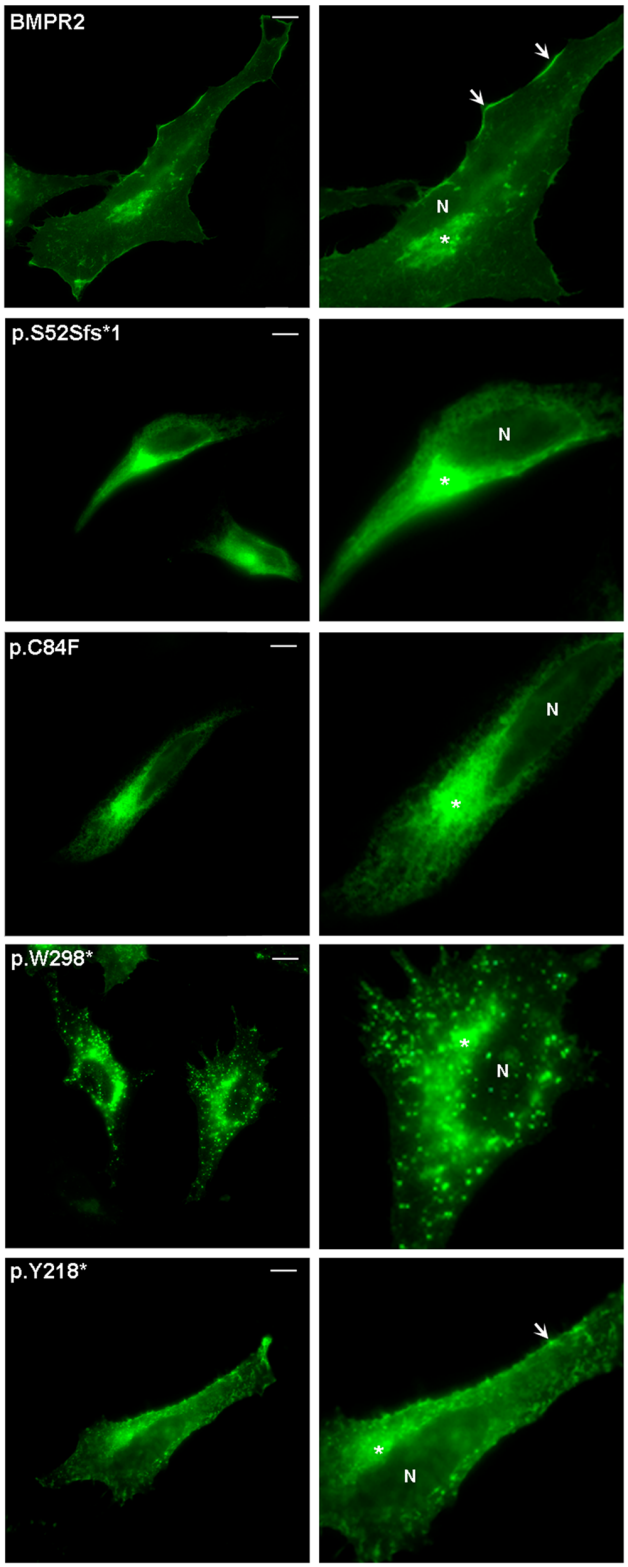
Representative photomicrograph showing subcellular localization of the BMPR2 wild-type and those mutants which pattern of expression is altered. All these constructs are GFP-tagged at the C-terminus. After 24 h post-transfection, HELA cells were fixed and mounted in Fluoromount-F. Images were visualized using a Leica TCS SP2 confocal system. Arrows indicates plasma membrane location. Asterisk indicates perinuclear region. The scale bar is 10 μm.

**Figure 6:**
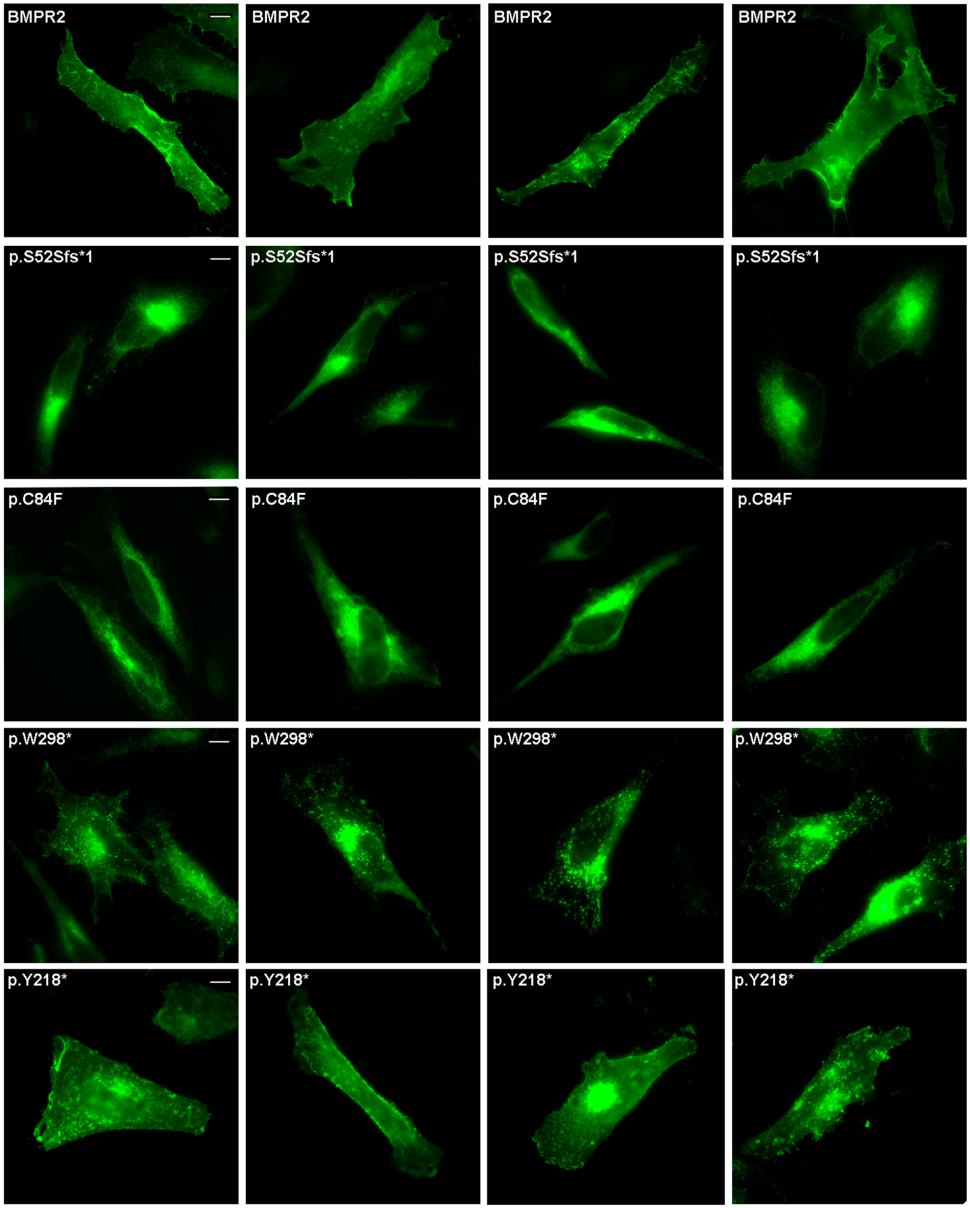
Photomicrograph example of the subcellular localization, in HELA cells, of the BMPR2 wild-type and those mutants which pattern of expression is altered. All these constructs are GFP-tagged at the C-terminus. After 24 h post-transfection, HELA cells were fixed and mounted in Fluoromount-F. Images were visualized using a Leica TCS SP2 confocal system. The scale bar is 10 μm.

**Figure 7:**
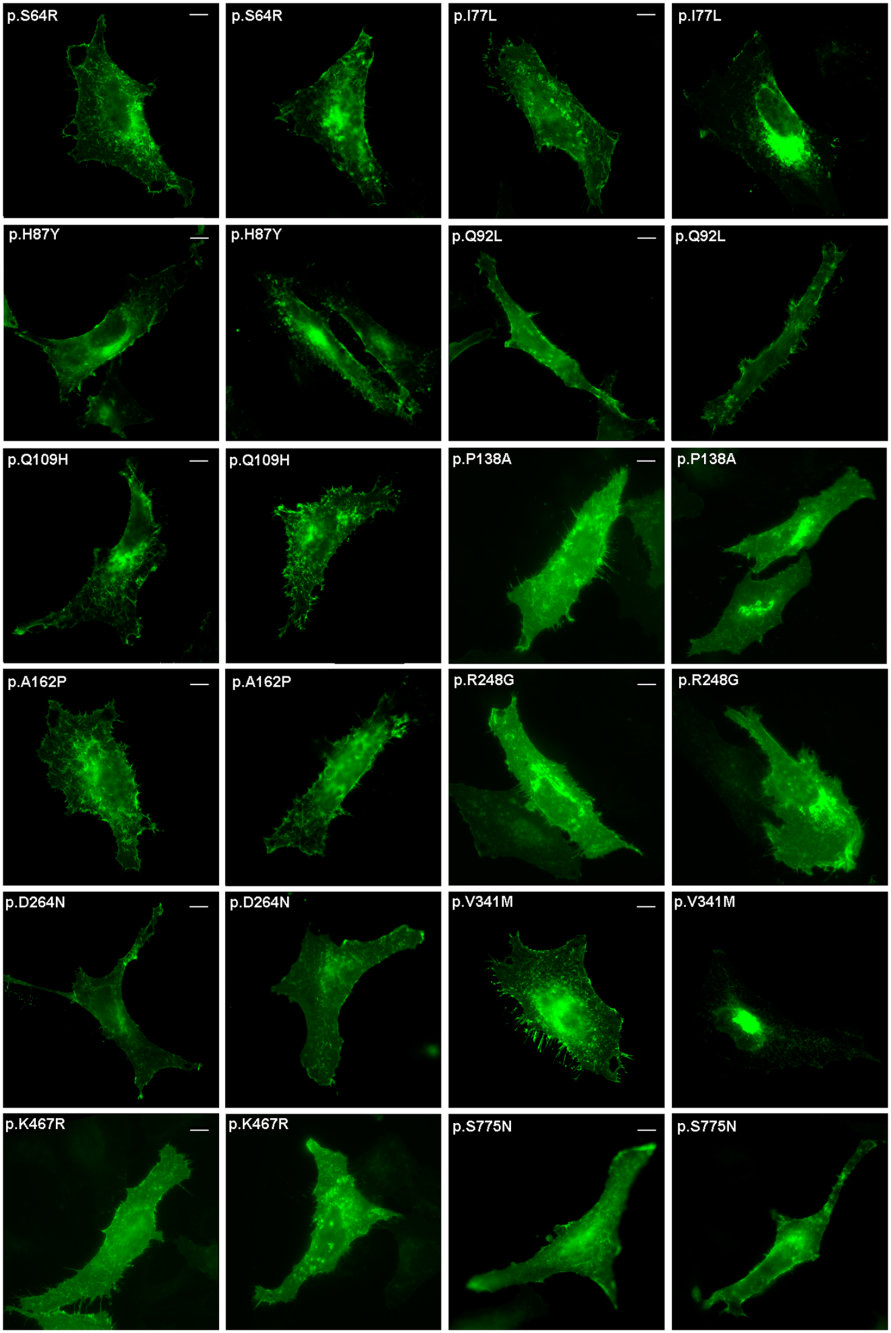
Representative photomicrograph showing subcellular localization of the BMPR2 mutants which pattern of expression is not altered. All these constructs are GFP-tagged at the C-terminus. After 24 h post-transfection, HELA cells were fixed and mounted in Fluoromount-F. Images were visualized using a Leica TCS SP2 confocal system. The scale bar is 10μm.

To determine the possible effect of the clinical mutations on the subcellular localization of the BMPR2 protein, we performed immunofluorescence assays. Our results indicated that in HeLa cells, the wild-type BMPR2 showed a plasma membrane and perinuclear localization (Fig. 5 and Fig. 6). However, one frameshift mutation, c.156_157delTC (p.S52Sfs*1), one nonsense mutation, c.893G > A (p.W298*), and one missense mutation, c.251G > T (p.C84F), show an abnormal cytoplasmatic pattern (Fig. 5). The c.654T > A (p.Y218*) mutation displayed a subcellular localization similar to that of wild-type, although some cells show a different punctate pattern of expression at the plasma membrane, compared to the wild-type BMPR2 protein (Fig. 5 and Fig. 6). The remaining missense mutations analyzed (c.190A > C, c.229A > T, c.259G > T, c.275A > T, c.327G > C, c.412 C > G, c.484 G > C, c.742 A > G, c.790 G > A, c.1021 G > A, c.1400 A > G and c.2324 G > A), mimic the pattern of the wild-type BMPR2 protein (Fig. 7).

### Luciferase assay

**Figure 8:**
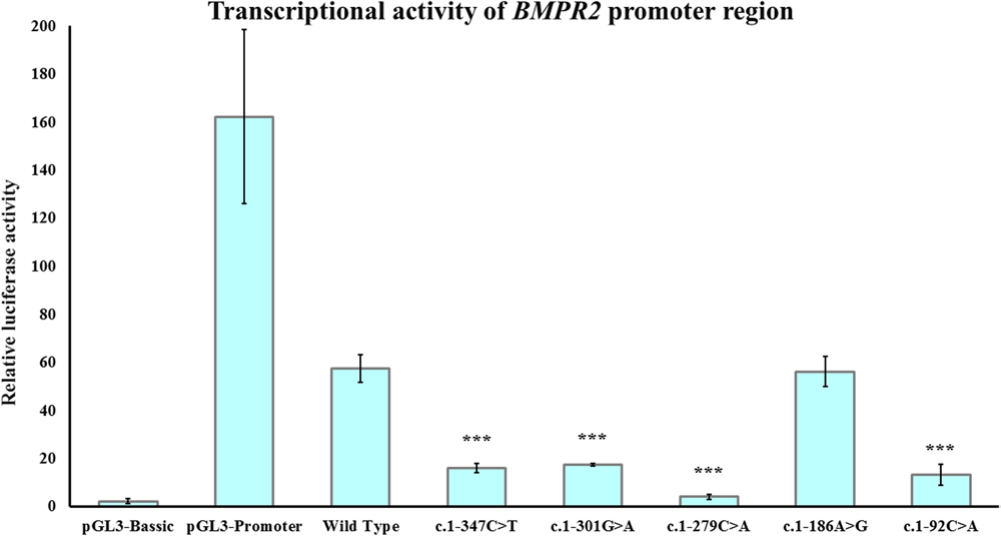
Transcriptional activity of the 5′UTR region of the *BMPR2* gene containing different mutations (c.1-347C > T, c.1-301G > A, c.1-279C > A, c.1-186A > T and c.1-92C > A). The pGL3-Basic vector containing no promoter element was used as negative control and the pGL3-Promoter vector fused with SV40 promoter element was used as positive control. c.1-347C > T, c.1-301G > A, c.1-279C > A, and c.1-92C > A mutations produces a decreased transcriptional activity of the 5′UTR region of the BMPR2 gene.

We observed statistically significant differences for 4 out of the 5 mutations in the gene transcriptional activity by luciferase assays. Three mutations (c.1-347 C > T, c.1-301 G > A and c.1-92 C > A) showed a decreased transcriptional activity that ranges between 70–77% (p < 0.0001) compared to wild-type levels. Remarkably, the c.1-279 C > A mutation produced the greatest decrease in the *BMPR2* transcriptional activity: 93% (p < 0.0001) (Fig. 8). The c.1-186 A > T change led to a decrease of about 2% with no statistical significance (p = 0.495) (Fig. 4). These pathogenic mutations (c.1-347 C > T, c.1-301 G > A. c.1-279 C > A and c.1-92 C > A) were found in 5 out of 55 patients included in this study.

All pathogenic mutations found in the *BMPR2* gene in our clinical series are summarized in Table 4.

### Association with clinical features and hemodynamic parameters

We classify 13 nucleotide variants found in 16 of 55 patients (29%) as pathogenic, according to the results obtained in the previously described assays. These mutations include synonymous, missense and nonsense mutations located in coding regions and changes located in the 5′UTR region. All of them have been described for the first time by our group, in this paper or in Pousada *et al*.^10^, except the c.1-301G > A (rs116154690) mutation located in the 5′UTR region.

We compared clinical features and hemodynamic parameters among patients with pathogenic changes in the *BMPR2* gene and without (Table 1). We found statistically significant differences in systolic pulmonary artery pressure (sPaP; p = 0.042) and 6 minutes walking test (6MWT; p = 0.041): patients carrying a *BMPR2* mutation presented with a higher values for this parameter than non-carriers. In addition, individuals harboring mutations had a significantly lower age at diagnosis (p = 0.040). Besides, patients with mutations seemed to have worse response to phosphodiesterase-5-inhibitors (p = 0.010). The presence of mutations was unrelated to gender (p = 0.360), mPaP (p = 0.266), PVR (p = 0.553) and cardiac index (CI) (p = 0.588). Clinical or pathogenic mutations were seen in 10 patients with IPAH and 6 patients with APAH without statistical differences (p = 0.137), clinical and hemodynamic parameters did not show significant differences between IPAH and APAH.

## Discussion

The characterization of functional relevance of variants found in human diseases is being a challenge. The identification of a causal mutation is essential for molecular diagnosis and clinical management of many genetic disorders. However, there are still difficulties in distinguishing which variants may cause or contribute to disease. Some articles^24, 25^ highlight that mutations directly affecting splice sites or altering potential regulatory elements are more prevalent. Current knowledge clearly indicates that, besides their protein coding potential, altered sequences can play an important role in RNA splicing. The functional characterization of these type of mutations will help in developing bioinformatics tools that allowed us to predict the causative effect of a particular mutation^24, 25^.

We hypothesized that variants located in the exonic region of *BMPR2* gene could lead to alterations which affect the proper splicing process or cause an abnormal protein subcellular localization. In addition, mutations in the 5′UTR region could be associated with a decrease in the transcriptional activity. The analyzed variants show a high clinical interest since all of them have been identified in heterozygous state and most of these variants represent the only genetic variant found in the in some patients of the cohort included in this study. Patients were clinically evaluated in detail and the functional analysis of these variants could help us to better understand the low penetrance associated with *BMPR2* mutations.

After mutational analysis of exonic and flanking regions of *BMPR2* gene in patients with PAH, several mutations were predicted to affect the splicing process. However, it is difficult to discriminate between neutral and pathogenic variants, based on splice site conservation and bioinformatics predictions exclusively^10, 26^. Thus, it is necessary to perform specific analyses to ascertain their pathogenicity and their possible involvement in the mRNA processing.

Twenty-two variants were detected and subsequently analyzed by *in silico* tools. Only 9 of them were predicted to render a shorter protein^27, 28^, either due to the deletion of some base pairs (c.412C > G, c.633A > G, c.1400A > G), to the deletion of a whole exon (c.251G > T, c.835G > T, c.981T > C), to a frameshift mutation which cause a premature stop codon (c.156_157delTC) or nonsense mutations (c.654T > A, c.893G > A). These mutations are located in exonic regions and have been shown to produce the elimination or creation of new splice sites^27, 28^. New cryptic dinucleotide sequences have been used for those mutations deleting some base pairs. Mutation c.412C > G generates a GG acceptor site, Sharma *et al*.^29^ perform several experiments with a GG change in the acceptor splice site of Cystic Fibrosis (CF) gene that generate an alternative splice site^29^. Mutation c.633A > G, is a T-to-C change in the invariant GT dinucleotide donor splice site modifying the cryptic splice site as described for mutation p.Q1291H in CF disease^30^. Finally, GA dinucleotide in the donor splice site is generated for c.1400A > G mutation, as previously described like a rare exception, in the *FGFR* gene family^31, 32^. However, several splice sites that do not follow the GT-AG rule have been reported^29–34^.

For synonymous mutations, we obtained an altered splicing pattern in 3 out of 7 mutations (c.633A > G, c.835G > T, c.981T > C), using a minigene study approach. This result highlights the importance of carrying out functional studies since, to date, the pathogenicity of synonymous variants has been disregarded^35^. The synonymous and other mutations under study (c.654T > A, c.893G > A, c.1400A > G) were located in the serine/threonine kinase domain, characterized by the presence of highly conserved regions^4, 10, 13, 14, 36^. These synonymous mutations alter the mRNA processing and lead to a shorter serine/threonine kinase domain. Giving the important role of this ATP-binding domain, any structural changes could avoid the interaction between SMADs proteins, inhibiting the TGF-β signaling pathway^10, 13, 37–39^. The remaining mutations associated with alterations in the splicing process are located in the N-terminal (c.156_157delTC and c.251G > T) and in the transmembrane (c.412C > G) domains. It is possible that nonsense and missense mutations that result in a premature stop codon could be degraded by nonsense mediated decay (NMD). Conversely, the mutations located in the transmembrane domain could yield a weaker anchorage of the BMPR2 protein, affecting its stability. Mutations in transmembrane domain of the *BMPR2* gene have been reported to trigger important physiological effects^39–42^.

Nevertheless, since the *BMPR2* gene is expressed in vascular endothelial cells, which are hardly accessible tissues, we could not perform functional analysis in specific tissues. It would also be interesting to analyze their impact in animal models and to check how these mutations could modify the phenotype *in vivo*. The splicing mutations represent more than 9% of published changes in PAH^4^, but experimental analyses could increase this percentage^19, 27, 42^. Cooper *et al*. have estimated that 1.6% of missense substitutions affect the mRNA processing^43^. Additional functional analyses are necessary to establish the mechanism underlying disease since not all the mutations associated with splicing alterations are classified as such after *in silico* analysis and vice versa. In this sense, minigene assay has been reported as a good approach to evaluate possible splicing alterations related to all these variants^19, 27, 28, 42, 44, 45^.

Otherwise, subcellular localization studies showed that the wild-type BMPR2 protein is located both in plasma membrane and perinuclear cell region, as has been reported by other authors^46–50^. The c.251G > T (p.C84F) missense mutation showed a diffuse cytoplasmic expression pattern which is similar to that observed for other clinical mutations affecting cysteines in the extra cellular domain^51–54^. Instead, according to minigene assays, the c.251G > T variant generates a frameshift mutation (p.C84Ffs*12). The p.S52Sfs*1 and p.W298* variants, which produce a truncated protein, are also mimicking a cytoplasmic expression pattern. However, one nonsense mutation (p.Y218*) behaves similar to the BMPR2 wild-type protein, although a different punctuate pattern at the plasma membrane is produced. These truncated form lacking transmembrane domain would probably lead to a mislocalization of the protein from plasma membrane. It has been reported that not only mutations located in N-terminal or ligand binding domains, but also mutations producing a premature stop codon, could be associated with alterations in protein localization^41, 51, 52^. These aberrant proteins could be retained in the endoplasmic reticulum (ER) and activate the ER quality control (ERQC)^53, 54^. Those species retained in ER tend to dimerize with themselves and the wild-type protein, exerting a dominant negative functional effect^53, 54^. The transduction signal mediated by BMPR2 also might be affected, inhibiting TGF-β pathway^55^. It is known that BMPR2 interacts with CAV1 (caveolin-1) in several cell types. In vascular smooth muscle cells this interaction regulates BMPR2 signaling, with an associated loss of BMPR2-dependent SMAD phosphorylation by decreasing the association between BMPR2 and BMPR1^4, 55–58^, and a down-regulation of other genes such as *SMADs* when levels of *CAV1* are decreased^46^. Furthermore, patients carrying mutations responsible for alterations in subcellular localization, show high levels of endothelin-1 (ET-1) and reduced levels of nitric oxide (NO)^55, 58^. Down-regulation of BMPR2 stimulates the production of ET-1 and reduces the production of NO^53, 57^. For this reason, we cannot discard the idea that mutants leading to an altered subcellular localization of BMPR2 could cause an endothelial imbalance of these two genetic modifiers. The aforementioned alterations and interactions at cellular level could be important in the context of homeostasis, providing an additional explanation for the variety of PAH phenotypes.

It has been described, by Girerd *et al*.^47^, that mutations located in the BMPR2 cytoplasmic tail could have a role in the development of PAH disease despite not affecting SMAD signaling pathway^47, 59^. Likewise, we have identified a variation located in the BMPR2 cytoplasmic tail (c.2324G > A)^58^ which does not involve any alteration in subcellular localization, confirming the trafficking to the cell surface. Little is known about the function of the cytoplasmic tail, but several studies confirm the ability of these mutations to activate SMAD signaling^47^. Additional functions have been described for this domain such as regulation of p38 and p42/44 MAPK and the interaction with LIMK, c-Src, Tctex and TRB3^47^.

Furthermore, 5′UTR variants have been related to several pathologies^21, 59–61^. However, a recent study suggests that mutations in this region only represent a second hit for the development of PAH and can lead to an earlier onset of symptoms and more severe clinical course, modifying its penetrance^26, 62–64^. Patients carrying mutations in the 5′UTR region, despite not having any other alteration in the studied genes^10, 26^, seem to present a more severe phenotype. All constructions, except c.1-186A > T, showed a decreased transcriptional activity, which could down-regulate BMPR2 expression and weaken Smad1/5 signaling^21, 65^. Thus, this could prevent BMPR1/BMPR2 complex binding and subsequent phosphorylation, thereby avoiding ligand binding^65, 66^. For this reason the translocation to the nucleus could be inhibited, with full or partial reduction of gene expression pattern. These changes can affect cell homeostasis by impairing the binding sites of transcription factors binding sites^61, 67–72^.

We have detected 30 total variants in the *BMPR2* gene. Of those, we have confirmed some functional effect in 13 of them. Besides, for c.275A > T, c.742A > C, c.790G > A and c.1021G > A mutations, we not detect any difference with wild-type regarding to splicing and subcellular localization analysis. Likewise, due to the absence or low frequency of these changes in dbSNP, ESP6500, ExAc and CSVS databases (<0.00001), its location in the serine/threonine kinase domain except for the first variant, and the classification of pathogenic by multiple software we consider them as VUS. After *in vitro* analysis, clinical mutations were detected in 29% of patients. This is, to our knowledge, the study with highest rate for BMPR2 mutations in which a functional analysis has been performed.

After genotype-phenotype correlation analysis, we found significant statistical differences for age at diagnosis, sPaP, 6MWT and response to treatment^65, 73, 74^. Remarkably, patients with nonsense mutations present a less severe phenotype than patients with other pathogenic mutations. This could be explained by other mechanisms instead of NMD or perhaps a dominant negative effect^75^. As suggested in mice, other pathways should take place in these cases in order to minimize the impact of *BMPR2* mutations^76, 77^.

This study does not have enough power to clarify the different treatment effect related to the presence of mutations. Perhaps the worst response found in carriers of mutations may be due to a greater impact on cell proliferation in the media and layers intima of the pulmonary arteries due to the deterioration of the pathways regulated by *BMPR2* gene. It would be very interesting to design prospective studies to determine which drugs would be most effective in patients with mutations in *BMPR2* gene and other genes involved in the development of PAH.

Our study confirms that the *BMPR2* gene is a leading player in the pathogenesis of PAH. An exhaustive analysis of this gene shows that a significant percentage of patients have some potentially pathogenic variant. The *in vitro* expression analysis is a good approach to determine the effect of the variants on mRNA processing, especially in those cases where the genes involved are expressed in tissues of difficult access. It is important to highlight the importance of investigating synonymous mutations and mutations located in the UTR regions, which can act as causal variants of disease as well.

The pathophysiology of PAH shows a complex mechanism in which the TGF-β pathway plays a decisive role in the development of the disease, and a heterogeneous constellation of genetic arrangements could be responsible for the pulmonary vascular remodeling.

## Material and Methods

### Patients and samples

Fifty-five patients with idiopathic or associated PAH (group 1 of the new classification of Nize)^6^ followed in our Pulmonary Arterial Hypertension Unit were enrolled. Cardiac catheterization was performed using the latest consensus diagnostic criteria of the ERS-ESC (mean resting pulmonary pressure ≥25 mmHg, PAWP <15 mmHg) in all cases^78^. PAH was considered idiopathic after exclusion of all the possible clinical causes associated with the disease. Clinical history included use of drugs, especially appetite suppressants, and screening for connective tissue and hepatic diseases. The study also included serology for HIV (human immunodeficiency virus), autoimmunity, thoracic CT scan (computerized tomography scan) and echocardiography. Patients with chronic lung disease that could be related to PAH, were excluded. Patients were considered responders to treatment when they met the following criteria: improvement of at least one functional class or maintenance in functional class II, decreased proBNP, 10% increase in the distance walked on the 6 minutes test, no hospital admissions related PAH and no need for new specific treatment for PAH. Samples from fifty individuals provided by the Complejo Hospitalario Universitario de Vigo, were used as controls.

Patients signed an informed consent and the Autonomic Ethics Committee approved the study (Galician Ethical Committee for Clinical Research; *Comité Autonómico de Ética da Investigación de Galicia - CAEI de Galicia*) followed the clinical-ethical practices of the Spanish Government and the Helsinki Declaration.

### Identification and analysis of mutations in *BMPR2* gene

Genomic DNA was extracted from leukocytes isolated from venous blood using the FlexiGene DNA Kit (Qiagen, Germany) according to the manufacturer’s protocol. We performed a Polymerase Chain Reaction (PCR) with 50 ng of genomic DNA from each patient and control in a MJ MiniTM Gradient Thermal Cycler (Bio-Rad, Hercules, California, USA), using GoTaq® Green Master Mix (Promega Corporation, Madison, Wisconsin, USA), which contained Taq DNA polymerase, dNTPs, MgCl_2_ and reaction buffer. Amplification conditions were as follows: 95 °C for 5 min, 35 cycles of 95 °C for 30 s, 55 °C for 30 s, 72 °C for 30 s and a final extension of 72 °C for 10 min. Forward and reverse primers, described by Deng Z *et al*.^75^ were used to amplify 5′UTR and coding region for *BMPR2* gene, and also intronic junctions. PCR products were separated by electrophoresis through 2% agarose gels containing ethidium bromide to confirm the PCR products, in a Sub-Cell GT chamber (Bio-Rad, Hercules, California, USA). The PCR products were then purified using the ExoSAP-IT kit (USB Corporation, Cleveland, Ohio, USA) and sequenced with the BigDye Terminator version 3.1 Cycle Sequencing Kit (Applied Biosystems, California, USA) in a GeneAmp PCR System 2700 (Applied Biosystems, California, USA). The PCR products were precipitated and finally analyzed in an ABI PRISM 3100 genetic analyzer (Applied Biosystems, California, USA). Sequences were aligned to the reference Ensembl DNA sequence [ENST00000374580]. All detected changes were confirmed by a second independent PCR reaction and were identified in both forward and reverse strands.

### In silico analysis

To predict whether a rare missense variant was deleterious, we used the combined results of four different computer algorithms: The Polymorphism Phenotyping Program (PolyPhen-2; available at http://genetics.bwh.harvard.edu/pph/)^79^, Pmut (available at http://mmb2.pcb.ub.es:8080/PMut/)^80^, Sorting Intolerant from Tolerant (SIFT; available at http://sift.jcvi.org)^81^ and MutationTaster2 software (available at http://www.mutationtaster.org/)^82^. We also analyzed intronic, synonymous, missense, nonsense and frameshift indels changes to predict whether those changes could potentially affect splicing process, by creating or eliminating donor/acceptor splice sites, with Neural Network SPLICE (*NNSplice*) 0.9 version from the Berkeley Drosophila Genome Project (http://fruitfly.org:9005/seq_tools/splice.html)^83^, *NetGene2* (http://www.cbs.dtu.dk/services/NetGene2)^84^, *Splice View* (http://bioinfo.itb.cnr.it/oriel/splice-view.html)^84^ and Human Splicing Finder (*HSF*) 2.4.1 version (http://www.umd.be/HSF)^85^. Finally, alterations in binding sites of transcription factors in 5′UTR region of *BMPR2* gene were evaluated with MatInspector software (available at https://www.genomatix.de/online_help/help_matinspector/matinspector_help.html).

### Design of minigene constructs

To perform minigene assay, we generated fragments containing the exon where the mutation was located and 150–200 bp of flanking intronic regions. We amplified them by PCR from normal genomic DNA using primers described in Table 5 and High Fidelity Phusion polymerase (Finnzymes, Espoo, Finland), and these fragments were subcloned into the pSPL3 vector (Invitrogen Corporation, Carlsbad, CA) at the *Xho*I/*Nhe*I restriction sites (New England Biolabs, Ipswich, Massachusetts, USA) using T4DNA Ligase (Invitrogen Corporation, Carlsbad, CA) to generate a construct with wild-type fragments. The cloning was confirmed by PCR with primers 5′-CATGCTCCTTGGGATGTTGAT-3′ (*forward*) and 5′-ACTGTGCGTTACAATTTCTGG-3′ (*reverse*).

**Table 5:** Primer sequences for minigene fragments in the *BMPR2* gene.

Nucleotide change (Amino acid change)	Primers	Size	Tª
**c.156_157delTC** (p.S52Sfs*2)	F 5′ → 3′: AAGAATCTCGAGGAATTCATGAACAGAAGAACG R 5′ → 3′: AAGAATGCTAGCCCTCGAAAAGTGCTGGAATTA	514bp	58 °C
**c.190A** > **C** (p.S64R)	F 5′ → 3′: AAGAATCTCGAGGAATTCATGAACAGAAGAACG R 5′ → 3′: AAGAATGCTAGCCCTCGAAAAGTGCTGGAATTA	514 bp	58 °C
**c.229A** > **T** (p.I77L)	F 5′ → 3′: AAGAATCTCGAGGAATTCATGAACAGAAGAACG R 5′ → 3′: AAGAATGCTAGCCCTCGAAAAGTGCTGGAATTA	514 bp	58 °C
**c.251G** > **T** (p.C84F)	F 5′ → 3′: AAGAATCTCGAGCCATGAAATGTCTTTGGTATC R 5′ → 3′: AAGAATGCTAGCCTACGCCCGGCTAATTTTTTA	582 bp	59 °C
**c.259G** > **T** (p.H87Y)	F 5′ → 3′: AAGAATCTCGAGCCATGAAATGTCTTTGGTATC R 5′ → 3′: AAGAATGCTAGCCTACGCCCGGCTAATTTTTTA	582 bp	59 °C
**c.275A** > **T** (p.Q92L)	F 5′ → 3′: AAGAATCTCGAGCCATGAAATGTCTTTGGTATC R 5′ → 3′: AAGAATGCTAGCCTACGCCCGGCTAATTTTTTA	582 bp	59 °C
**c.327G** > **A** (p.Q109Q)	F 5′ → 3′: AAGAATCTCGAGCCATGAAATGTCTTTGGTATC R 5′ → 3′: AAGAATGCTAGCCTACGCCCGGCTAATTTTTTA	582 bp	59 °C
**c.327G** > **C** (p.Q109H)	F 5′ → 3′: AAGAATCTCGAGCCATGAAATGTCTTTGGTATC R 5′ → 3′: AAGAATGCTAGCCTACGCCCGGCTAATTTTTTA	582 bp	59 °C
**c.412C** > **G** (p.P138A)	F 5′ → 3′: AAGAATCTCGAGCCATGAAATGTCTTTGGTATC R 5′ → 3′: AAGAATGCTAGCCTACGCCCGGCTAATTTTTTA	582 bp	59 °C
**c.484G** > **C** (p.A162P)	F 5′ → 3′: AAGAATCTCGAGACTTGGTGTTTTAGTGTTCC R 5′ → 3′: AAGAATGCTAGCGAAAGGGGTAGTGACTGATAA	521 bp	57 °C
**c.600A** > **C** (p.L200L)	F 5′ → 3′: AAGAATCTCGAGCCAGAATTTGGCTTTCATGC R 5′ → 3′: AAGAATGCTAGCGTTCACCTATGTTCCTAGTG	468 bp	58 °C
**c.633A > G** (p.R211R)	F 5′ → 3′: AAGAATCTCGAGCATCAGCCATACTAGAACAG R 5′ → 3′: AAGAATGCTAGCGCTGGAATTACAGATGTGTG	591 bp	58 °C
**c.637C > A** (p.R213R)	F 5′ → 3′: AAGAATCTCGAGCATCAGCCATACTAGAACAG R 5′ → 3′: AAGAATGCTAGCGCTGGAATTACAGATGTGTG	591 bp	58 °C
**c.654T** > **A** (p.Y218*)	F 5′ → 3′: AAGAATCTCGAGCATCAGCCATACTAGAACAG R 5′ → 3′: AAGAATGCTAGCGCTGGAATTACAGATGTGTG	591 bp	58 °C
**c.742A** > **G** (p.R248G)	F 5′ → 3′: AAGAATCTCGAGCATCAGCCATACTAGAACAG R 5′ → 3′: AAGAATGCTAGCGCTGGAATTACAGATGTGTG	591 bp	58 °C
**c.790G** > **A** (p.D264N)	F 5′ → 3′: AAGAATCTCGAGCATCAGCCATACTAGAACAG R 5′ → 3′′: AAGAATGCTAGCGCTGGAATTACAGATGTGTG	591 bp	58 °C
**c.835G** > **T** (p.V278V)	F 5′ → 3′: AAGAATCTCGAGCATCAGCCATACTAGAACAG R 5′ → 3′: AAGAATGCTAGCGCTGGAATTACAGATGTGTG	591 bp	58 °C
**c.893G** > **A** (p.W298*)	F 5′ → 3′: AAGAATCTCGAGGTTTAAATTCCCCTTTCCATC R 5′ → 3′: AAGAATGCTAGCGAGTTTTACTCAGCTATCAAG	560 bp	58 °C
**c.981T** > **C** (p.P327P)	F 5′ → 3′: AAGAATCTCGAGAGTGGCAGCATGTTTGTTAG R 5′ → 3′: AAGAATGCTAGCGGTCTCGAACTCTTTACCTT	549 bp	58 °C
**c.1021G** > **A** (p.V341M)	F 5′ → 3′: AAGAATCTCGAGAGTGGCAGCATGTTTGTTAG R 5′ → 3′: AAGAATGCTAGCGGTCTCGAACTCTTTACCTT	549 bp	58 °C
**c.1400A** > **G** (p.K467R)	F 5′ → 3′: AAGAATCTCGAGTTAGGATTTCCAAATGTGCC R 5′ → 3′: AAGAATGCTAGCGATTTGTGGCATTAGGCAAC	412 bp	57 °C
**c.1467G** > **A** (p.E489E)	F 5′ → 3′: AAGAATCTCGAGTCACCTTTTGAGCATGTTCC R 5′→ 3′: AAGAATGCTAGCCAGATTTCATCTTGCACTTG	549 bp	57 °C

F: Forward; R: Reverse.

bp: Base pair.

### Design of pEGFP-N1 constructs

To perform cellular localization studies, we generated wild-type and mutated BMPR2 constructs. The human full-length cDNA of BMPR2, obtained from the MGC Human BMPR2 Sequence-Verified cDNA (ID: 6149698; GE Healthcare Dharmacon Inc, Lafayette, CO, USA), was subcloned into the mammalian expression vector pEGFP-N1, with the GFP at the C-terminal end (Clontech, Mountain View, CA, USA), with High Fidelity Phusion polymerase (Finnzymes, Espoo, Finland) and with primers 5′-GGAAGTCGACCATGACTTCCTCGCTGCAGCG-3′ (*forward)* and 5′-GCGCCTCGAGCATTTCACAGACAGTTCATTCC-3′ (*reverse)*.

### Design of luciferase constructs

The Dual-Luciferase Reporter Assay System (Promega, USA) was chosen to establish the activity of 5′UTR region in *BMPR2* gene. The 539 bp human *BMPR2* 5′UTR region was amplified by PCR from normal genomic DNA using the primers 5′-AAGAATGCTAGCGGAAGCACCGAAGCGAAAC-3′ (forward) and 5′-AAGAATCTCGAGCCCTGGGCCAGCCAAGAAT-3′ (reverse). PCR was performed with High Fidelity Phusion polymerase (Finnzymes, Espoo, Finland) and the resultant fragments were subcloned into the pGL3-Basic vector (Promega, Madison, Wisconsin, USA) at the NheI/XhoI restriction sites (New England Biolabs, Ipswich, Massachusetts, USA), using T4DNA Ligase (Invitrogen Corporation, Carlsbad, CA) to generate a construct with wild-type 5′UTR region driving firefly luciferase expression gene. The fragments were inserted with the same orientation, and their sequences were confirmed by DNA sequencing using RVprimer3 and GLprimer2 (Promega, Madison, Wisconsin, USA). The pGL3-Basic vector containing no promoter element was used as negative control and the pGL3-Promoter vector (Promega, Madison, Wisconsin, USA) fused with SV40 promoter element was used as positive control.

### Directed Mutagenesis

Mutagenesis of the wild-type fragments to generate mutant minigenes for each of the identified variants, mutagenesis of the wild-type cDNA of *BMPR2* gene construct to performed cellular localization studies and mutagenesis of the wild-type 5′UTR region of *BMPR2* gene (firefly luciferase) to generate mutant constructs for each change, was performed by using QuikChange XL-II Site-Directed Mutagenesis Kit (Stratagene, Agilent, Santa Clara, CA, USA) according to the manufacturer’s protocol. The specific primer sequences containing the nucleotide changes for minigene constructs and cellular localization are indicated in Table 6 and the primer sequences to luciferase constructs are available in Table 7. The p.S52Sfs*1, p.Y218* and p.W298* constructs for cellular localization were generated by subcloning the sequence of interest, amplified from pEGFP-N1: BMPR2 plasmid, into the SalI and BamHI restriction sites of the pEGFP-N1 vector. Plasmids were sequenced after site-directed mutagenesis to confirm their authenticity and to rule out additional nonspecific changes.

**Table 6:** Primer sequences used to perform mutagenesis for each minigene and subcellular localization in the *BMPR2* gene.

Nucleotide change (Amino acid change)	Primers
**c.156_157delTC** (p.S52Sfs*2)	F 5′ → 3′: GGATAGGTGAGAGTAGAATCTCATGAAAATGGGACAATATTATG R 5′ → 3′: CATAATATTGTCCCATTTTCATGAGATTCTACTCTCACCTATCC
**c.190A > C** (p.S64R)	F 5′ → 3′: GGGACAATATTATGCTCGAAAGGTCGCACCTGCTATGG R 5′ → 3′: CCATAGCAGGTGCAACCTTTCGAGCATAATATTGTCCC
**c.229A > T** (p.I77L)	F 5′ → 3′: GGGAGAAATCAAAAGGGGACTTAAATCTTGTAAAACAAGGC R 5′ → 3′: GCCTTGTTTTACAAGATTTAAGTCCCCTTTTGATTTCTCCC
**c.251G > T** (p.C84F)	F 5′ → 3′: CTTTTTTGTATTCATATTGATTTATAGGATTTTGGTCTCACATTGGAG R 5′ → 3′: CTCCAATGTGAGACCAAAATCCTATAAATCAATATGAATACAAAAAAG
**c.259G > T** (p.H87Y)	F 5′ → 3′: ATCTTGTAAAACAAGGATGTTGGTCTTACATTGGAGATCCC R 5′ → 3′: GGGATCTCCAATGTAAGACCAACATCCTTGTTTTACAAGAT
**c.275A > T** (p.Q92L)	F 5′ → 3′: TCACATTGGAGATCCCCTAGAGTGTCACTATGAAG R 5′ → 3′: CTTCATAGTGACACTCTAGGGGATCTCCAATGTGA
**c.327G > A** (p.Q109Q)	F 5′ → 3′: CACTCCTCCCTCAATTCAAAATGGAACATACCGTTTC R 5′ → 3′: GAAACGGTATGTTCCATTTTGAATTGAGGGAGGAGTG
**c.327G > C** (p.Q109H)	F 5′ → 3′: CACTCCTCCCTCAATTCACAATGGAACATACCGTTTC R 5′ → 3′: GAAACGGTATGTTCCATTGTGAATTGAGGGAGGAGTG
**c.412C > G** (p.P138A)	F 5′ → 3′: CACCTCCTGACACAACAGCACTCAGTAAGTAAAGT R 5′ → 3′: ACTTTACTTACTGAGTGCTGTTGTGTCAGGAGGTG
**c.484G > C** (p.A162P)	F 5′ → 3′: TGGCATCAGTCTCTGTATTACCTGTTTTGATAGTTGCC R 5′ → 3′: GGCAACTATCAAAACAGGTAATACAGAGACTGATGCCA
**c.600A > C** (p.L200L)	F 5′→ 3′: CCAACAGTTTCAGATTATCGAGATCAAGAGAGGGTTCGG R 5′ → 3′: CCGAACCCTCTCTTGATCTCGATAATCTGAAACTGTTGG
**c.633A > G** (p.R211R)	F 5′ → 3′: CAGCTGATTGGCCGGGGTCGATATGGAGC R 5′ → 3′: GCTCCATATCGACCCCGGCCAATCAGCTG
**c.637C > A** (p.R213R)	F 5′ → 3′: AGCTGATTGGCCGAGGTAGATATGGAGCAGTATAT R 5′ → 3′: ATATACTGCTCCATATCTACCTCGGCCAATCAGCT
**c.654T > A** (p.Y218*)	F 5′ → 3′: CGAGGTCGATATGGAGCAGTATAAAAAGGCTCCTTGG R 5′ → 3′: CCAAGGAGCCTTTTTATACTGCTCCATATCGACCTCG
**c.742A > G** (p.R248G)	F 5′ → 3′: AATTTTATCAACGAAAAGAACATTTACGGAGTGCCTTTGATGGAAC R 5′ → 3′: GTTCCATCAAAGGCACTCCGTAAATGTTCTTTTCGTTGATAAAATT
**c.790G > A** (p.D264N)	F 5′ → 3′: CCCGCTTTATAGTTGGAAATGAGAGAGTCACTGCA R 5′ → 3′: TGCAGTGACTCTCTCATTTCCAACTATAAAGCGGG
**c.835G > T** (p.V278V)	F 5′ → 3′: CGCATGGAATATTTGCTTGTTATGGAGTACTATCCCAATGT R 5′ → 3′: ACATTGGGATAGTACTCCATAACAAGCAAATATTCCATGCG
**c.893G > A** (p.W298*)	F 5′ → 3′: ATTTAAGTCTCCACACAAGTGACTAGGTAAGCTCTTGC R 5′ → 3′: GCAAGAGCTTACCTAGTCACTTGTGTGGAGACTTAAAT
**c.981 T > C** (p.P327P)	F 5′ → 3′: ATCCAAACAGATCATTATAAACCCGCAATTTCCCATCGAGATTTAAA R 5′ → 3′: TTTAAATCTCGATGGGAAATTGCGGGTTTATAATGATCTGTTTGGAT
**c.1021G > A** (p.V341M)	F 5′ → 3′: GAGATTTAAACAGCAGAAATGTCCTAATGAAAAATGATGGAACCTG R 5′ → 3′: CAGGTTCCATCATTTTTCATTAGGACATTTCTGCTGTTTAAATCTC
**c.1400A > G** (p.K467R)	F 5′ → 3′: CTTACCAGGCTATTTTCCTTCCAGGCTTCTGGGAA R 5′ → 3′: TTCCCAGAAGCCTGGAAGGAAAATAGCCTGGTAAG
**c.1467G > A** (p.E489E)	F 5′ → 3′: GTTGGGACCAGGATGCAGAAGCTCGGCTTA R 5′ → 3′: TAAGCCGAGCTTCTGCATCCTGGTCCCAAC

F: Forward; R: Reverse.

**Table 7:** Primer sequences for mutagenesis in 5′UTR region of the *BMPR2* gene.

Nucleotide change	Primers
**c.1-347C > T**	F 5′ → 3′: CTGGATATGTTTTCTCCCAGACTTGGATATTTTTTTGATATCGTG
	R 5′ → 3′: CACGATATCAAAAAAATATCCAAGTCTGGGAGAAAACATATCCAG
**c.1-301G > A**	F 5′ → 3′: ACGAGGGAAATAATTTGGAGGATTTCTTCTTGGCTCC
	R 5′ → 3′: GGAGCCAAGAAGAAATCCTCCAAATTATTTCCCTCGT
**c.1-279C > A**	F 5′ → 3′: TCTTCTTGGCTCCCTGATTTCCCCACAGACATG
	R 5′ → 3′: CATGTCTGTGGGGAAATCAGGGAGCCAAGAAGA
**c.1-186A > T**	F 5′ → 3′: GGGAGAGAAATGAAGGGTATTTCTGCAGCGGCATG
	R 5′ → 3′: CATGCCGCTGCAGAAATACCCTTCATTTCTCTCCC
**c.1-92C > A**	F 5′ → 3′: GGGCAGGATCAGTCCAAGGGAGAGAAGACG
	R 5′ → 3′: CGTCTTCTCTCCCTTGGACTGATCCTGCCC

F: Forward; R: Reverse.

### Cell culture and transfections

We used COS-7 cells for minigene assay, HELA cells for subcellular localization studies and COS-1 cells for luciferase assay. All cell lines were cultured in DMEM (Gibco, Grand Island, USA), supplemented with 10% Fetal Bovine Serum (FBS; Gibco, Grand Island, USA), 1% L-Glutamine (Lonza, Basel, Switzerland) and 1% penicillin/streptomycin (Lonza, Basel, Switzerland), in a humidified atmosphere at 37 °C with 5% CO_2_. COS-7 cells with 80–90% confluency at passage three were transiently transfected by using 2 µg of pSPL3 construct (wild-type and mutant construct) per well. HELA cells were cultured in six-well plates on glass coverslips. On the other hand, COS-1 cells with 80–90% confluency were transfected by using 2 µg of pGL3-Bassic construct (with 5′UTR region of *BMPR2* gene, wild-type and mutant) per well and pGL3-Promoter vector as negative control. COS-1 cells were co-transfected with 20 ng of pRL-CMV vector (control Renilla plasmid). COS-7 transfection was performed using Lipofectamine 2000 (Invitrogen Corporation, Carlsbad, CA, USA) in a 2:5 ratio of μg of plasmid DNA to μl of Lipofectamine. Hela and COS-1 transfection was performed using Fugene HD (Promega, Madison, Wisconsin, USA), in a 2:5 and 1:3 ratio of μg of plasmid DNA to μl of Fugene HD, respectively, and following the manufacturer’s instructions.

### Minigene Assay

COS-7 cells were incubated during 36 h after transfection and all experiments were performed in duplicate. RNA extraction was performed with the Nucleospin RNA II Kit (Macherey-Nagel, Düren, Germany) according to the manufacturer’s protocol. RT-PCR was carried out with geneAmp Gold RNA PCR Core Kit (Applied Biosystems, California, USA) and the resulting cDNA was amplified with High Fidelity Phusion polymerase (Finnzymes, Espoo, Finland) with primers 5′-TCTGAGTCACCTGGACAACC-3′ (*forward*) and 5′-ATCTCAGTGGTATTTGTGAGC-3′ (reverse), which anneal with the SD6 and SA2 exons in pSPL3 vector. Amplification conditions were as follows: 98 °C for 30 s, 30 cycles of 98 °C for 10 s, 58 °C for 30 s, 72 °C for 30 s and finally, 72 °C for 7 min. The PCR products were separated by electrophoresis on 2% agarose gel to analyze changes in the transcript pattern, and sequenced with the BigDye Terminator version 3.1 Cycle Sequencing Kit (Applied Biosystems, California, USA) to identify changes in splicing.

### Subcellular Localization Assay

For subcellular localization studies, HELA cells were fixed 24 h post-transfection in 4% (w/v) paraformaldehyde in phosphate-buffered saline (PBS) for 15 min. After several washes with PBS, samples were mounted in Fluoromount-F (Souther Biotech, Birmingham, AL, USA). Images were visualized using a Leica TCS SP2 confocal system. In order to determine the pattern of localization for each mutant, a blind assessment by two researchers was performed using an average of 40 cells for each construct.

### Luciferase Assay

Cells were harvested at 36 h after transfection. Luciferase assays were performed with the Dual Luciferase Assay System (Promega, Madison, Wisconsin, USA) on a 2104 EnVision Luminometer (PerkinElmer, Waltham, Massachusetts, USA). Cells were lysed in 500 µL of passive lysis buffer (PLB buffer) at room temperature for 30 min. For dual luciferase assay, 20 µL of lysate was aliquoted into a 96-well plate for measuring firefly luciferase (100 µL of LAR II) and renilla luciferase (100 µL of stop and glow buffer) activity. Firefly values were divided by Renilla values to normalize for fluctuations in plated cells and transfection efficiency. All experiments were repeated twice in triplicate and we analyzed two different constructs for the wild-type and the mutations.

### Statistical analysis

Values are expressed as mean ± SD (standard deviation). Differences between groups were examined for statistical significance using Student’s t-test. Chi-square test was used to compare clinical and hemodynamic variables among genotypes (variables were categorized according to the best cut off point by ROC curve). These correlations were analyzed by the Spearman test. Probability values less than 0.05 were considered statistically significant. Statistical analyses were performed using SPSS for Windows (v19.0).
